# Plasma atherogenic indices are independent predictors of slow coronary flow

**DOI:** 10.1186/s12872-021-02432-5

**Published:** 2021-12-20

**Authors:** Abdulmecit Afsin, Hakan Kaya, Arif Suner, Kader Eliz Uzel, Nurbanu Bursa, Yusuf Hosoglu, Fethi Yavuz, Ramazan Asoglu

**Affiliations:** 1 Department of Cardiology, Adiyaman Training and Research Hospital, Adiyaman, Turkey; 2 Department of Cardiology, Faculty of Medicine, Adiyaman University, Adiyaman, Turkey; 3 Department of Statistics, Faculty of Science, Hacettepe University, Ankara, Turkey

**Keywords:** Coronary slow flow, Frame count, Atherogenic index of plasma, Castelli risk indices, Coronary intervention, Cardiovascular risk factors, Coronary artery

## Abstract

**Background:**

Although the pathophysiology of coronary slow flow (CSF) has not been fully elucidated, emerging data increasingly support potential role for subclinical diffuse atherosclerosis in the etiology of CSF. We aimed to investigate relationship between atherogenic indices and CSF.

**Methods:**

130 patients with CSF diagnosed according to Thrombolysis in Myocardial Infarction (TIMI)-frame count (TFC) method and 130 controls who had normal coronary flow (NCF) were included in this retrospective study. Atherogenic indices (atherogenic index of plasma [AIP], Castelli risk indices I and II [CRI-I and II]) were calculated using conventional lipid parameters.

**Results:**

The logistic regression analyses demonstrated that AIP (OR, 5.463; 95% confidence interval [CI], 1.357–21.991; *p* = 0.017) and CRI-II (OR, 1.624; 95% CI, 1.138–2.319; *p* = 0.008) were independent predictors of CSF. Receiver operating characteristic analysis showed that the optimal cutoff value to predict the occurrence of CSF was 0.66 for AIP (sensitivity, 59%; specificity, 73%; area under curve [AUC], 0.695; *p* < 0.001) and 3.27 for CRI-II (sensitivity, 60%; specificity, 79%; AUC, 0.726; *p* < 0.001).

**Conclusions:**

AIP and CRI-II levels were independent predictors of CSF. Prospective studies in larger cohorts of patients may elucidate the role of atherogenic dyslipidemia in the pathophysiology of CSF.

## Introduction

Coronary slow flow (CSF) is a microvascular disorder characterized by the slow entry of radiopaque contrast agent into distal vascular structures in the presence of normal or near-normal epicardial coronary arteries during coronary angiography [1]. Although there is no clear consensus regarding the pathophysiology of CSF, diffuse atherosclerosis, inflammation, increased platelet aggregability, increased microvascular tone, microvascular and endothelial dysfunction have been suggested to contribute to its pathogenesis [2]. Multiple studies to define the demographic characteristics of patients with CSF have shown that male sex, smoking, and decreased high-density lipoprotein cholesterol (HDL-C) level are more common in these patients [3–5].

Atherogenic dyslipidemia, which comprises the concurrence of increased serum triglyceride (TG), apolipoprotein B, and small dense low-density lipoprotein cholesterol (LDL-C) levels along with decreased HDL-C level, plays a major role in the genesis of atherosclerotic plaques [6]. Atherogenic dyslipidemia has been implicated in the pathogenesis of endothelial dysfunction, microvascular coronary dysfunction, and atherosclerosis. [7]. Oxidized LDL inhibits endothelium dependent vasodilation by impairing the activity of nitric oxide synthase and contributes to the atherosclerotic plaque formation [8]. HDL protects endothelial cells against the damaging effects of LDL and improves endothelial cell function [9]. Studies have demonstrated that hypertriglyceridemia impairs endothelial function through several mechanisms [10]. TG also induces atherosclerosis via the production of proinflammatory cytokines, coagulation factors, and fibrinogen [11]. The atherogenic index of plasma (AIP) is a relatively novel indicator of atherogenicity calculated as log_10_ (TG/HDL-C) [12]. Previous studies have demonstrated that, in comparison to simple lipid parameters, the atherogenic coefficient (AC; Non-HDL-C/HDL-C) and Castelli’s risk indices I and II (total cholesterol [TC]/HDL-C and LDL-C/HDL-C, respectively) had stronger correlations with cardiovascular disease and better predictive capability for cardiovascular events [13–15].

Emerging data increasingly support potential role for endothelial dysfunction and subclinical diffuse atherosclerosis in the etiology of CSF. There is insufficient data in the literature on the relationship between atherogenic indices and CSF. For this reason, the present study was performed to investigate the relationships of lipid profile and atherogenic indices with CSF.

## Methods

### Study population and design

**Fig. 1:**
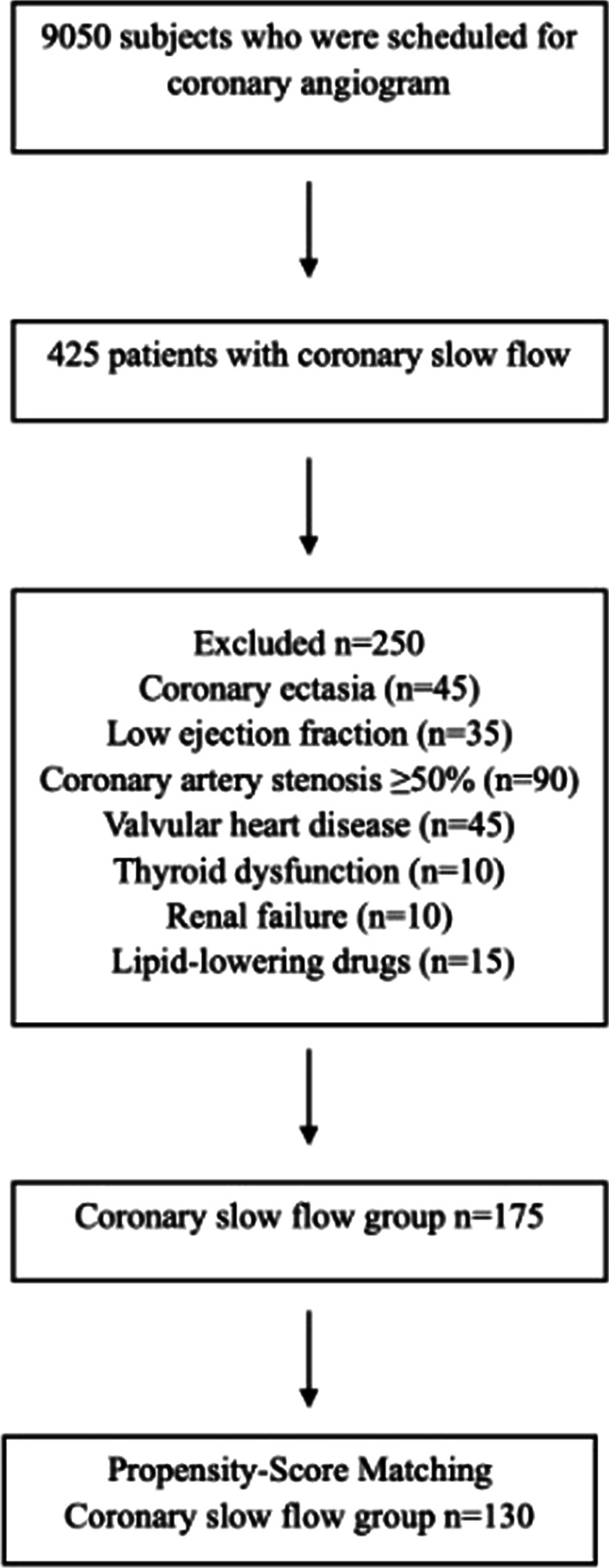
Diagram shows the selection of the study groups

We retrospectively analyzed the data of 9050 patients who had undergone diagnostic coronary angiography between January 2017 and August 2020 at Adiyaman UniversityAffiliated Hospital, with the indications of detection of ischemia in the exercise treadmill testing or myocardial perfusion scintigraphy after admission with stable angina pectoris or unstable angina pectoris. In total, 425 patients with CSF who did not have significant stenosis in the left main coronary artery, the other three major coronary arteries, or their side branches above 2.0 mm on coronary angiography were identified. After exclusion criteria and propensity score matching, the study group consisted of 130 patients (1.5%) with CSF. 130 individuals with normal coronary flow (NCF) in coronary angiography were included in the study as a control group. Selection of the study group is summarized in Fig. 1. All participants provided written informed consent for coronary angiography. The Adiyaman University Clinical Research and Ethics Committee approved the protocol (2021/02-10–16/02/2021), and the study was performed in accordance with the principles of the Declaration of Helsinki.

Serum TC level > 200 mg/dL was regarded as indicative of dyslipidemia. Hypertension was defined as blood pressure ≥ 140/90 mm/Hg or receiving antihypertensive treatment. Diabetes mellitus was defined as fasting blood glucose level ≥ 126 mg/dL or known diabetes mellitus diagnosis. Smoking status was regarded as positive for current smokers and for those who had quit smoking within the past 1 year with a smoking history of > 10 pack-years. Patients were excluded from the study if they had a previous history of acute myocardial infarction, previous percutaneous coronary intervention or coronary artery bypass graft surgery, and/or had coronary ectasia. Patients were also excluded from the study if they had coronary artery stenosis ≥ 50%, cerebrovascular disease, renal failure, left ventricular systolic dysfunction (left ventricular ejection fraction [LVEF] ≤ 50), moderate to severe valvular heart disease, congenital heart disease, cardiomyopathies (dilated, restrictive, hypertrophic), hematological disease, thyroid dysfunction, and/or inflammatory diseases, and if they used lipid-lowering drugs or lacked complete clinical data. Demographic, clinical, and laboratory data of the participants were obtained from the medical records of our hospital.

### Coronary angiography and TIMI frame count (TFC)

All coronary angiographies were performed either from the femoral or radial access using the standard technique (Siemens Axiom Artis zee 2011; Siemens Healthcare, Erlangen, Germany). Iopromide contrast medium (Bayer Pharma AG, Berlin, Germany) was used in all patients. Images of the coronary arteries were acquired in the right and left oblique planes, as well as the cranial and caudal angles, at 15 frames per second (fps). Two cardiologists who were blinded to the patients’ demographic and clinical features assessed all angiograms to define CSF. The TIMI frame count (TFC) method developed by Gibson et al. [16] was used for quantitative measurement of coronary blood flow. The first frame was defined as the frame in which the contrast agent reached the ostium and the coronary artery was first visualized, and the last frame was defined as the frame in which the contrast agent was first visualized at the distal point. The distal bifurcation (i.e., “moustache”) of the left anterior descending artery (LAD), the distal bifurcation of the longest branch of the left circumflex artery (LCX), and the level at which the first lateral branch originated from the posterolateral artery of the right coronary artery (RCA) were defined as the end points. Because the calculated TFC value for LAD was substantially higher than the RCA and LCX counts, the LAD frame count was divided by the average of the numbers obtained from LCX and RCA counts to allow standardization, producing a constant coefficient of 1.7. The corrected TFC (cTFC) for LAD was calculated by dividing the LAD TFC by 1.7.

Mean reference values of 36 ± 1 TFC for LAD, 22.2 ± 4 TFC for LCX, and 20.4 ± 3 TFC for RCA were reported for the filling of coronary arteries [16]. In the present study, patients in whom the measured TFC values were greater than or equal to two standard deviations of the mean in at least one coronary artery were considered to have CSF. The mean TFC for each patient and control participant was calculated by dividing the sum of the TFCs of LAD, LCX, and RCA by 3.

### Laboratory examination

Laboratory findings were collected from the hospital database. Before the angiography, blood samples were collected for complete blood count analyses, following a 12 h overnight fasting. Plasma TC, TG, LDL-C, HDL-C, fasting glucose, creatinine levels were analyzed using the Architect c8000 Chemistry System (Abbott Diagnostics, USA) commercial kits. LDL-C was calculated via direct LDL-C assays. Then, AIP was determined by the base 10 logarithm of the ratio of the TG level to HDL-C level. The other indices used in this study were calculated as follows: non-HDL-C = (TC-HDL-C), AC = (non-HDL-C/HDL-C), CRI-I = (TC/HDL-C) and CRI-II = (LDL-C/HDL-C).

Complete white blood (WBC) counts, including neutrophil and lymphocyte counts, were measured using an automated hematology analyzer CELL-DYN Ruby (Abbott Diagnostics, Abbott Park, IL, USA) and expressed as × 1.000 cells/mm^3^. Hemoglobin and platelet count were also calculated. Neutrophil to lymphocyte ratio (NLR) was calculated by dividing the neutrophil count to the lymphocyte count, and platelet to lymphocyte ratio (PLR) was calculated as the number platelets divided by the lymphocyte count. Transthoracic echocardiographic evaluation was performed for all patients by using Vivid 5 Pro (General Electric, Horten, Norway) brand echocardiography device. LVEF was assessed using Simpson's method [17].

### Statistical analysis

The sample size of the study group was determined with 0.80 power and medium effect size using power analysis in R environment (R Core Team, 2020). More than 25% of this sample size (n = 260) was included in the study because of the possibility of using nonparametric tests and can be missing values. To reduce the bias when selecting the participants to CSF and NCF groups propensity score with the nearest neighbor method and 1:1 allocation ratio was used. While calculating the score, the gender and smoking status of participants took consideration. However, there was a total of 150 subjects in the NCF group and 175 subjects in the CSF group that fit the criteria. These limited numbers restricted the flexibility to get similar groups very much while selecting subjects into the groups.

All analyses were performed using SPSS, version 23 (IBM Corp., Chicago, IL, USA) and R, version 4.0.5 (R Core Team, 2020) software. Continuous variables were presented as mean ± standard deviation or median (quartile deviation), and categorical variables were presented as numbers and percentages. Kolmogorov–Smirnov test was used to determine whether the continuous variables were distributed normally or not. Independent sample t-test or Mann–Whitney U-test was used to compare continuous variables. Categorical variables were compared within the study group using chi-squared tests. Receiver operating characteristic (ROC) curve analysis was performed to find a cut-off value for AIP and CRI-II according to Youden’s J index. Multiple logistic regression analysis with forward variable selection was used to determine the predictors of CSF. Hosmer–Lemeshow test was used to evaluate model fit. The odds ratio (OR) and 95% confidence interval (CI) were calculated for each independent variable. In all analyses *p* < 0.05 was considered statistically significant.

## Results

In total, 130 patients with CSF and 130 control participants with NCF were included the study. The mean ages of the two groups were 54.05 ± 9.61 years and 54.82 ± 8.78 years, respectively. Both groups showed male predominance (80% and 56%, respectively). Table 1 shows the demographic and clinical characteristics of the study population. Age, hypertension status, diabetes mellitus status, and dyslipidemia status were similar between groups. The proportion of male sex and current smokers were significantly higher in the CSF group than in the NCF group (*p* < 0.001). The WBC, NLR, PLR, neutrophil cell count, platelet count, LDL-C, TG, non-HDL-C, AIP, AC, CRI-I, and CRI-II values were significantly higher in the CSF group than in the control group. Furthermore, the HDL-C level and lymphocyte cell count were significantly lower in the CSF group than in the control group. There were no statistically significant differences between the two groups in terms of other laboratory parameters, TC, and LVEF (all, *p* > 0.05).

**Table 1:** Demographic and laboratory findings of the study population

Characteristics	CSF (n = 130)	NCF (n = 130)	*p*
Gender (male), n (%)	104 (80%)	73 (56%)	**< 0.001**
Age (years)	54.05 ± 9.61	54.82 ± 8.78	0.501
BMI, kg/m^2^	29.6 ± 3.5	28.9 ± 4.1	0.425
Smoking, n (%)	79 (61%)	36 (28%)	**< 0.001**
Hypertension, n (%)	52 (40%)	57 (44%)	0.615
Diabetes mellitus, n (%)	52 (40%)	37 (29%)	0.067
Dyslipidemia, n (%)	76 (59%)	67 (52%)	0.319
Haemoglobin, g/dL	13.9 ± 1.5	13.9 ± 1.6	0.909
White blood cell count, (× 10^3^/μL)	8.67 ± 2.09	8.10 (1.55)	**0.029**
Neutrophil cell count, (× 10^3^/μL)	5 (1.18)	4.67 (0.85)	**0.006**
Lymphocyte cell count, (× 10^3^/μL)	2.11 ± 0.54	2.30 (0.47)	**0.008**
NLR	2.49 (0.48)	1.96 (0.55)	**< 0.001**
Platelet (10^3^/μL)	277 (58.25)	251 (38.75)	**< 0.001**
Mean platelet volume (fL)	8.43 ± 1.34	8.39 (0.63)	0.857
PLR	128.39 (32.44)	106.18 (24.43)	**< 0.001**
Fasting glucose, mg/dl	103.50 (15.25)	108.50 (16.13)	0.329
Creatinine, mg/dl	0.80 (0.12)	0.78 (0.08)	0.080
LV ejection fraction (%)	58.6 ± 4.1	57.0 ± 2.6	0.452
Total cholesterol, mg/dl	201.22 ± 34.32	197.50 (26.13)	0.411
HDL cholesterol, mg/dl	38.50 (5.13)	46 (7.50)	**< 0.001**
LDL cholesterol, mg/dl	130.95 ± 29.82	121.38 ± 33.53	**0.016**
Triglyceride, mg/dl	182.50 (58.88)	150 (50.37)	**< 0.001**
Non-HDL cholesterol, mg/dl	162.50 ± 34.39	151.74 ± 32.35	**0.010**
Atherogenic index of plazma	0.70 ± 0.22	0.53 ± 0.24	**< 0.001**
Castelli’s risk index I	5.43 ± 1.44	4.45 ± 0.98	**< 0.001**
Castelli’s risk index II	3.55 ± 1.13	2.74 ± 0.84	**< 0.001**
Atherogenic coefficient	4.43 ± 1.44	3.45 ± 0.98	**< 0.001**
ACEI/ARB, n (%)	40 (31%)	45 (35%)	0.569
Calcium canal blocker, n (%)	20 (15%)	22 (17%)	0.781
Beta-blocker, n (%)	35 (27%)	30 (23%)	0.432
Antiplatelet, n (%)	32 (24%)	25 (19%)	0.247

*ACEI* angiotensin-converting enzyme inhibitor, *ARB* angiotensin receptor blocker, *Atherogenic coefficient* non-HDL-C /HDL-C, *Atherogenic index of plasma* log TG/HDL-C, *BMI* body mass index, *Castelli’s risk index I* TC/HDL-C, *Castelli’s risk index II* LDL-C/HDL-C, *CSF* coronary slow flow, *Non-HDLc* TC-HDL-C, *HDL* High-density lipoprotein, *LDL* Low-density lipoprotein, *LV* left ventricular, *NCF* normal coronary flow, *NLR* neutrophil lymphocyte ratio, *PLR* platelet lymphocyte ratio. Bold indicates *p* value < 0.05 was considered significant

The corrected LAD-TFC, LAD-TFC, LCX-TFC, RCA-TFC, and mean TFC values were significantly higher in the CSF group than in the control group (all, *p* < 0.001). In addition, in 73% of the patients, CSF was observed in the LAD (Table 2).

**Table 2:** Thrombolysis in Myocardial Infarction (TIMI) frame counts of study population

	CSF (n = 130)	NCF (n = 130)	*p*
TFC (frame)			
LAD	40 (6.5)	14 (2)	**< 0.001**
Corrected LAD	23.53 (3.83)	8.24 (1.17)	**< 0.001**
LCX	18 (3.5)	9 (1.5)	**< 0.001**
RCA	20 (4.5)	10 (1.5)	**< 0.001**
Mean TFC	26 (2.34)	11 (1.21)	**< 0.001**
Distribution of coronary arteries relative to slow flow			
LAD, n (%)	95 (73%)		
LCX, n (%)	40 (31%)		
RCA, n (%)	66 (51%)		

*CSF* slow coronary flow, *Cx* left circumflex coronary artery, *LAD* left anterior coronary artery, *TFC* Thrombolysis in Myocardial Infarction frame counting, *NCF* normal coronary flow, *RCA* right coronary artery. Bold indicates *p* value < 0.05 was considered significant

The multivariate logistic regression analyses demonstrated that AIP (OR, 5.463; 95% CI, 1.357–21.991; *p* = 0.017), CRI-II (OR, 1.624; 95% CI, 1.138–2.319; *p* = 0.008), PLR (OR, 1.004; 95% CI, 1.000–1.008; *p* = 0.049), current smoking (OR, 3.063; 95% CI, 1.664–5.641; *p* < 0.001), and male gender (OR, 3.464; 95% CI, 1.746–6.875; *p* < 0.001) were independent predictors of CSF (Table 3).

**Table 3:** Logistic regression analysis to identify the predictors of CSF

	OR	95% CI	*p*
AIP	5.463	1.357–21.991	**0.017**
CRI-II	1.624	1.138–2.319	**0.008**
PLR	1.004	1.000–1.008	**0.049**
Sex (reference: female)	3.464	1.746–6.875	**< 0.001**
Smoking (reference: non-smoking)	3.063	1.664–5.641	**< 0.001**

*AIP* Atherogenic index of plazma, *CI* Confidence interval, *CSF* Coronary slow flow, *CRI-II* Castelli’s risk index II, *PLR* Platelet lymphocyte ratio. Bold indicates *p* value < 0.05 was considered significant

**Fig. 2:**
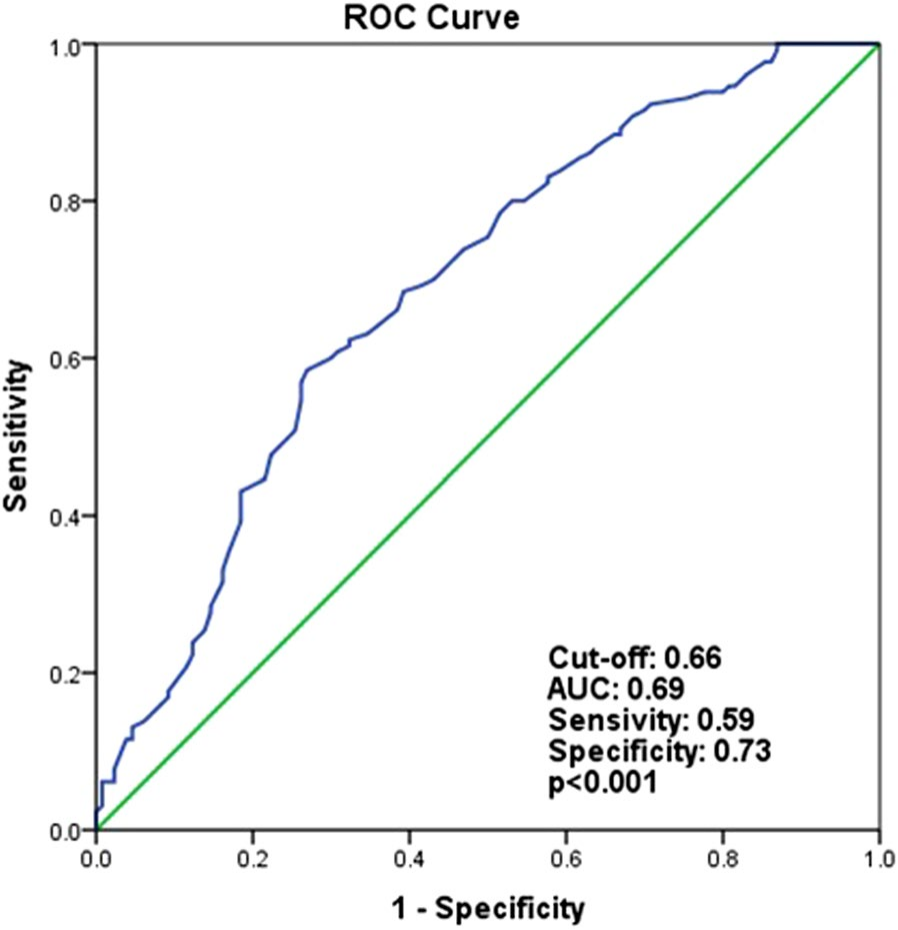
Receiver operating characteristics curve analysis to detect the best cut-off values of atherogenic index of plazma for differentiation between slow and normal coronary flows. *AUC* area under the curve

**Fig. 3:**
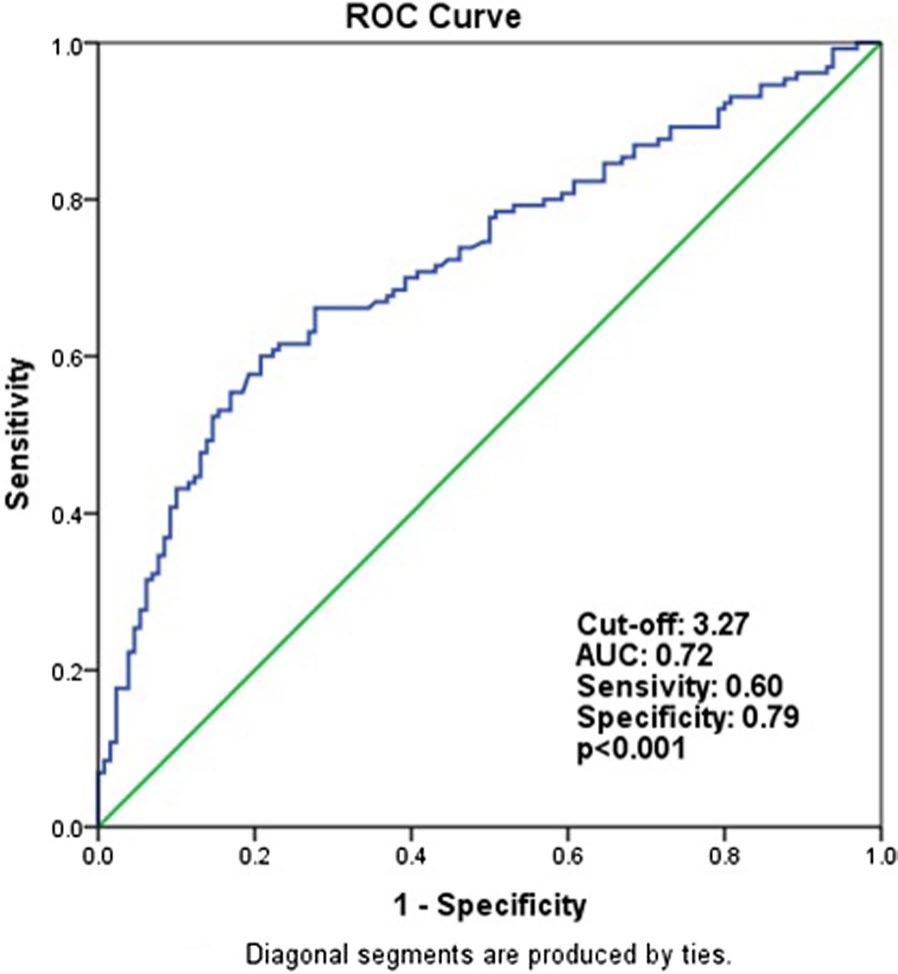
Receiver operating characteristics curve analysis to detect the best cut-off values of castelli risk indice II for differentiation between slow and normal coronary flows. *AUC* areaunder the curve

ROC curve analysis showed that the optimal cutoff value to predict the occurrence of CSF was 0.66 for AIP (sensitivity, 59%; specificity, 73%; area under the receiver operating characteristic curve, 0.695; *p* < 0.001) (Fig. 2). According to ROC analysis, the optimal cutoff value to predict the occurrence of CSF was 3.27 for CRI-II (sensitivity, 60%; specificity, 79%; area under the receiver operating characteristic curve, 0.726; *p* < 0.001) (Fig. 3).

## Discussion

This study was performed to investigate the relationships of CSF with traditional lipid parameters and atherogenic indices (e.g., AIP, AC, CRI-I, and CRI-II) that have been associated with increased cardiovascular risk. Atherogenic indices were higher and HDL-C values were lower in patients with CSF, compared with the control group. AIP, CRI-II, current smoking, and male sex were found to be an independent risk factor for CSF. In addition, LDL-C and non-HDL-C values were significantly higher in the CSF group compared to the control group.

In CSF, washout of the contrast agent is prolonged in the absence of any spasm, thrombus, dissection, and any stenosis that causes significant occlusion in epicardial coronary arteries. Although the incidence of CSF in diagnostic coronary angiography is not rare, its pathogenesis has not yet been well elucidated. Various mechanisms have been proposed for its etiology, including subclinical diffuse atherosclerosis. Cin et al. [18] examined the coronary arteries of 19 patients with CSF using fractional flow reserve and intravascular ultrasonography. They noted extensive calcification and diffuse intimal thickening along the vessel walls and atheroma plaques that did not cause lumen narrowing in patients with CSF, as well as diffuse atherosclerosis in the microvascular system and epicardial coronary arteries. Pekdemir et al. [19] reported diffuse intimal thickening and calcifications along epicardial arteries during coronary angiography examinations of patients with CSF. Ding et al. [20] reported that lipoprotein-associated phospholipase A2, which plays a role in inflammation and atherosclerosis in the vessel walls, was significantly and independently associated with the presence of CSF. Considering the data obtained from these studies, it may be reasonable to conclude that diffuse coronary atherosclerosis plays a role in the etiopathogenesis of CSF.

Increases in the levels of plasma very-low-density lipoprotein and small dense LDL-C, and reduced clearance of apolipoprotein B-containing particles from plasma have been identified in patients with atherogenic dyslipidemia [21]. Compared with other LDL subfractions, small dense LDL particles are more atherogenic because they are more susceptible to oxidative stress and can pass through the subendothelial space more easily due to their small diameter. Accordingly, they can stay in circulation longer and have less affinity for LDL receptors [22]. With regard to the origin of small dense LDL formation, Berneis et al. [23] proposed that TG-rich lipoproteins (e.g., very-low-density lipoprotein 1) are converted into small dense LDL after delipidation by hepatic lipase and lipoprotein lipase enzymes. The Framingham Heart Study showed that small dense LDL level is directly correlated with serum TG level and inversely correlated with serum HDL-C level in patients with metabolic syndrome [24]. There is increasing evidence that both predominance and elevated levels of small dense LDL-C play important roles in the initiation and progression of atherosclerosis, as well as increased risk of cardiovascular disease [22, 25, 26]. In a recent meta-analysis of 21 studies, Liou et al. [27] reported positive associations of small dense LDL level and cholesterol content of small dense LDL with the risk of coronary heart disease. These findings are supported by an increasing body of evidence in favor of the causal link between small dense LDL and coronary heart disease. However, because the test to measure small dense LDL is complex and costly, its measurement is unlikely to be applicable in routine clinical practice [28]. It was suggested that the TG/HDL-C ratio could be used as an indicator of LDL subfraction [29]. In addition, TG/HDL-C ratio increases systemic and vascular inflammatory processes by decreasing endothelial protective mechanisms, which leads to progressive coronary atherosclerosis [30].

AIP is regarded as an indirect indicator of small dense LDL-C [12]. Wang et al. [31] described a strong correlation between AIP and syntax score in patients with coronary heart disease. In a prospective observational study of women > 60 years old, a negative correlation between HDL-C concentration and AIP was founded, but observed a positive correlation between all-cause deaths and AIP, after adjusting for age, smoking, and statin therapy [32]. In a study conducted in 1059 patients with a history of acute coronary syndrome before the age of 35 years, the presence and severity of acute coronary syndrome were found to be independently associated with AIP, and these relationships were stronger than those of simple lipid parameters (i.e., TC, TG and LDL-C) [33]. TG/HDL-C ratio, AIP and CRI indices have predicted cardiovascular events better than traditional lipid profiles such as LDL-C and non-HDL-C [34].

To our knowledge, there have been no studies regarding the relationship between CSF and AIP. In the present study, we identified a positive correlation between AIP and TIMI frame count, which is regarded as an indicator of coronary flow reserve. The predictability of TIMI frame count by AIP supports the role of diffuse atherosclerosis in the pathophysiology of CSF. The results of the present study showed that AIP provided a reference for CSF severity. In addition, LDL-C and non-HDL-C, which are defined as the main indicator of atherogenic particles by current guidelines, were higher in the CSF group than in the control group. However, these lipid parameters were not found as predictive variables in the regression analysis.

CRI-II (LDL-C/HDL-C ratio) represents the proportion or relationship between the atherogenic and antiatherogenic lipoproteins. CRI-II was a more precise predictor for cardiovascular events than classic lipid parameters (i.e., TC, TG and LDL-C) used independently [13, 35]. Fujihara et al. [36] demonstrated that CRI-II was an independent predictor of coronary artery stenosis and vulnerable coronary plaque. Bleda et al. [37] showed that improvement of nitrite plasma levels, a marker of endothelial function, was associated with decreased total cholesterol/HDL-C values after statin treatment in patients with peripheral artery disease. Kıs et al. [38] reported that HDL-C/LDL-C ratio was associated with endothelial functions in patients with coronary artery disease. They showed that as the HDL-C/LDL-C ratio increased, flow-mediated dilatation increased. It has been suggested that while nitric oxide and endothelial progenitor cells were increased, LDL-C and inflammation markers (i.g., high sensitivity C-reactive protein, endothelin-1and interleukin-6) were decreased after atorvastatin treatment in patients with CSF [39]. In a study involving 54 patients with CSF, Kalaycı et al. [40] reported that TG/HDL-C ratio, CRI-I and II values were higher in the CSF group than in the control group. The authors stated that age, smoking and TG predict CSF. In the present study, CRI-II was an independent predictor of CSF which was different than Kalaycı et al. [40] investigation.

CSF has been reported to be more common in young male smokers. In our study, both smoking and male sex were independent risk factors for CSF. These results are consistent with previous studies. In their cross sectional study, Sanghvi et al. [41] reported that history of tobacco use was 45.5% and male sex was 62.5% in the CSF group. Furthermore, current smoking was an independent risk factor for CSF. In another study performed by Rao V et al. [4] in an Indian population, 66% of patients were males and 68% of patients were smokers in subject with CSF. In a prospective study involving 39 patients with CSF, Arbel et al. [3] reported that current smoking was the most significant variable related to CSF. Smoking association with CSF could be explained as follows: endothelial dysfunction, impaired endothelium-dependent coronary vasodilatation, increased microvascular resistance, increased oxidized LDL, and increase in mediators leading to atherosclerosis [3, 5, 42, 43].

There have been multiple investigations of the roles of inflammation and biomarkers that reflect the inflammatory state (e.g., white blood cells [overall and subtypes], acute phase reactants, adhesion molecules, and cytokines) in coronary artery disease, as well as their relationships to adverse events [44–47]. Aksan et al. [44] proposed that the inflammatory biomarker neutrophil gelatinase-associated lipocalin may be a useful marker and predictor for CSF. Turhan et al. [46] reported that the levels of plasma soluble adhesion molecules, such as intercellular adhesion molecule-1, vascular cell adhesion molecule-1, and E-selectin were elevated in patients with CSF. In addition, the biomarkers PLR [48] and NLR [49] are reportedly elevated in patients with CSF, compared with controls. These parameters have been significantly and independently associated with CSF. Our results were consistent with those of previous studies, in which NLR and PLR indices were higher in patients with CSF than in controls. PLR was only identified as predictive variable in regression analysis. Large platelets are more thrombotic, contain more dense granules, and are metabolically and enzymatically more active, compared with smaller platelets [50]. Their dense granules contain substances that are crucial mediators of coagulation, thrombosis, inflammation, and atherosclerosis [51]. In the present study, platelet values were higher in the CSF group, but there were no significant differences between the two groups in terms of MPV values.

## Study limitations

This study had several limitations. First, this was a single-center, retrospective observational study with a small sample size. Because of its retrospective design, inflammatory markers related to atherosclerosis (e.g., high-sensitivity C-reactive protein, interleukin-6, and adhesion molecules) were not studied, and a more detailed evaluation of the relationship between CSF and inflammation could not be performed. Apolipoprotein B and small dense LDL-C, which reflect the total atherogenic particle load better than LDL-C, were also not measured. Unfortunately, imaging modalities (e.g., intravascular ultrasonography or optical coherence tomography) could not be used, although these would have better demonstrated a potential relationship between CSF and subclinical diffuse atherosclerosis. Endothelial function tests such as pulse wave analysis and flow-mediated dilatation could not be performed. In addition, selection bias may have occurred during selection of the control group due to the retrospective study design, and some individuals with NCF in the control group may have had undetected microvascular dysfunction.

## Conclusions

The results of this study indicated that AIP and CR II are independent predictors of CSF. Prospective studies in larger cohorts of patients may elucidate the role of atherogenic dyslipidemia in the pathophysiology of CSF. The results may also provide additional support for the importance of lipid-lowering therapy in the management of patients with CSF.

## Data Availability

The datasets used and analysed during the current study are available from the corresponding author upon reasonable request.
